# The Role of M1/M2 Macrophage Polarization in Rheumatoid Arthritis Synovitis

**DOI:** 10.3389/fimmu.2022.867260

**Published:** 2022-05-19

**Authors:** Maurizio Cutolo, Rosanna Campitiello, Emanuele Gotelli, Stefano Soldano

**Affiliations:** Laboratory of Experimental Rheumatology and Academic Division of Clinical Rheumatology, Department of Internal Medicine and Specialties (DIMI), University of Genova, Istituto di Ricovero e Cura a Carattere Scientifico (IRCCS) San Martino Polyclinic Hospital, Genoa, Italy

**Keywords:** Macrophage polarization, Rheumatoid anhritis, Inflammation, Synovitis, bDMARD therapy

## Abstract

Innate and adaptive immunity represent a harmonic counterbalanced system involved in the induction, progression, and possibly resolution of the inflammatory reaction that characterize autoimmune rheumatic diseases (ARDs), including rheumatoid arthritis (RA). Although the immunopathophysiological mechanisms of the ARDs are not fully clarified, they are often associated with an inappropriate macrophage/T-cell interaction, where classical (M1) or alternative (M2) macrophage activation may influence the occurrence of T-helper (Th)1 or Th2 responses. In RA patients, M1/Th1 activation occurs in an inflammatory environment dominated by Toll-like receptor (TLR) and interferon (IFN) signaling, and it promotes a massive production of pro-inflammatory cytokines [i.e., tumor necrosis factor-α (TNFα), interleukin (IL)-1, IL-12, IL-18, and IFNγ], chemotactic factors, and matrix metalloproteinases resulting in osteoclastogenesis, erosion, and progressive joint destruction. On the other hand, the activation of M2/Th2 response determines the release of growth factors and cytokines [i.e., IL-4, IL-10, IL-13, and transforming growth factor (TGF)-β] involved in the anti-inflammatory process leading to the clinical remission of RA. Several subtypes of macrophages have been described. Five polarization states from M1 to M2 have been confirmed in *in vitro* studies analyzing morphological characteristics, gene expression of phenotype markers (CD80, CD86, TLR2, TLR4, or CD206, CD204, CD163, MerTK), and functional aspect, including the production of reactive oxygen species (ROS). An M1 and M2 macrophage imbalance may induce pathological consequences and contribute to several diseases, such as asthma or osteoclastogenesis in RA patients. In addition, the macrophage dynamic polarization from M1 to M2 includes the presence of intermediate polarity stages distinguished by the expression of specific surface markers and the production/release of distinct molecules (i.e., nitric oxide, cytokines), which characterize their morphological and functional state. This suggests a “continuum” of macrophage activation states playing an important role during inflammation and its resolution. This review discusses the importance of the delicate M1/M2 imbalance in the different phases of the inflammatory process together with the identification of specific pathways, cytokines, and chemokines involved, and its clinical outcomes in RA. The analysis of these aspects could shed a light on the abnormal inflammatory activation, leading to novel therapeutical approaches which may contribute to restore the M1/M2 balance.

## Introduction

Rheumatoid arthritis (RA) is a chronic systemic autoimmune inflammatory condition affecting approximately 1% of the population worldwide with considerable regional variation and an incidence rate higher in female than in male (1). Recognized as one of the most common autoimmune rheumatic diseases (ARDs) predominantly observed in the elderly population, RA is characterized by polyarticular synovitis at the level of small- and medium-sized joints, symmetrical joint swelling, tenderness, and redness as a result of the synovial lining layer inflammation, leading to joint damage and progressive disability (2–4). In this frame, multiorgan manifestations may arise during disease progression showing classical circadian rhythms (5).

Uncontrolled RA lowers life expectancy, and RA patients may have a roughly double average risk for developing malignancy and cardiovascular diseases (6). Although RA pathophysiology remains elusive, the presence of a complex interplay among genotype, epigenetic changes, and environmental factors underlying chronic inflammation is abundantly described (7, 8).

It is well established that among different risk factors, cigarette smoking, ozone exposure, and traffic-related air pollution are environmental elements significantly correlated to RA susceptibility, especially in those patients seropositive to rheumatoid factor (FR), anti-citrullinated peptide antibodies (ACPA), and anti-carbamylated protein antibodies.

Toxic components in smoke may enhance the activation of peptidylarginine deiminase (PAD) enzymes leading to a massive lung-protein citrullination. Additionally, smoke recalls antigen-presenting cells (APC), followed by T-helper-1 (Th1) activation, and finally anti-citrullinated peptide antibodies (ACPA)-specific B-cell memory formation (9, 10) .

A growing scientific interest is currently directed to highlight the role of intestinal microbiota and nutritional habits in RA patients (11, 12). In fact, diet may critically shape and alter the human gut microbiota composition, creating a “dysbiotic state,” which modulates the immune regulatory function and promotes a pro-inflammatory status (13). Of note, the extra virgin olive oil, a crucial component of Mediterranean diet, seems to reduce both presence and function of pro-inflammatory M1 macrophages and increase that of anti-inflammatory M2 macrophages (11, 12).

In recent years, the pathophysiological roles of innate immune system in RA have been investigated. In RA, the delicate balance among Th1/M1 and Th2/M2 system is lost giving way to an aberrant and uncontrolled Th1/M1 activation leading to organ damage (14). Astonishing steps have been made towards a better understanding of the central role of macrophages in RA chronic inflammation on-set and progression.

This review focus on the monocyte/macrophage contribution in RA pathogenesis primarily highlighting the immune-pathophysiological impact and imbalance of M1 and M2 macrophages and their precursors monocytes, and the identification of specific pathways, cytokines, and chemokines involved in mediating the abnormal inflammatory activation. Finally, the impact of current therapies that might contribute to reprogram macrophages, promoting their polarization from a pro-inflammatory M1 phenotype into an anti-inflammatory M2 phenotype as possible new strategy in the resolution of RA inflammatory process, is also analyzed.

## Circulating Monocytes in RA

Monocytes are circulating cells belonging to the mononuclear phagocytic system and known as the second line of defense in the innate immune system (15). Monocytes can play an important role in the initiation and maintenance of inflammation in the synovial tissue of RA patients: in fact, these cells are recruited from the circulation into the synovial tissue by chemotaxis through the interaction with fibroblast-like synoviocytes (FLSs) and other autoimmune cells (15).

**Figure 1:**
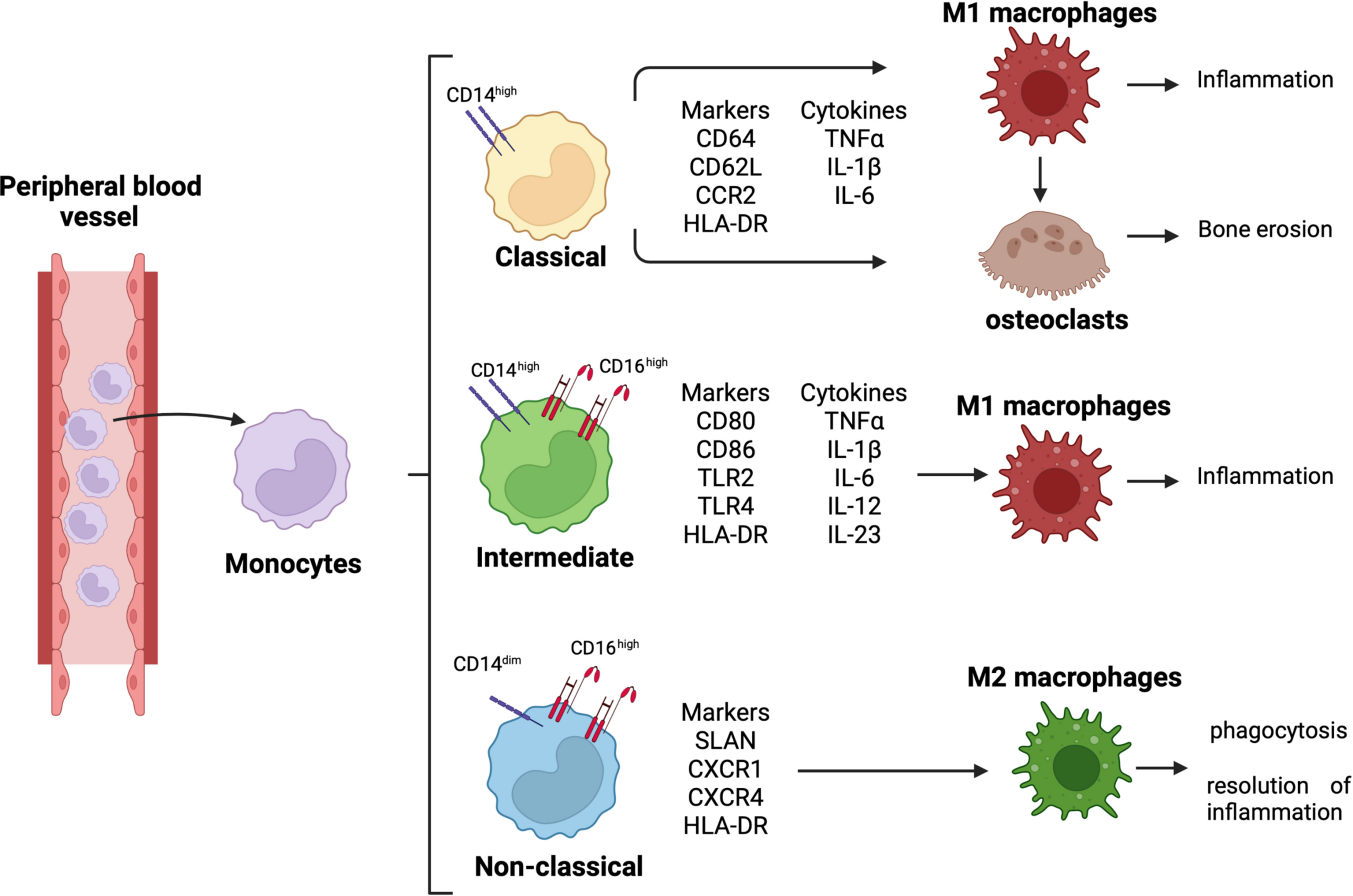
Monocyte differentiation and related role in RA pathogenesis. Differentiation of circulating monocytes in their three subsets, classical (CD14^high^), intermediate (CD14^high^CD16^high^), and non-classical (CD14^dim^CD16^high^) monocytes. Classical monocytes can differentiate into pro-inflammatory macrophages and osteoclasts, contributing to synovial tissue inflammation and bone erosion; intermediate monocytes differentiate into pro-inflammatory macrophages contributing to tissue inflammation; non-classical monocytes differentiate into anti-inflammatory macrophages promoting phagocytosis and resolution of inflammation.

Monocytes are classified into three subsets: classical monocytes (CD14^++^CD16^−^), intermediate monocytes (CD14^++^CD16^+^), and non-classical monocytes (CD14^dim^CD16^++^) (**Figure 1**) (16, 17). In RA synovial joints, classical monocytes seem to be the circulating precursors of osteoclasts involved in bone erosion (**Figure 1**) (18).

Moreover, the expression of CD14 and CD16 is upregulated on the monocyte cell membrane, and the percentage of intermediate monocyte subset is higher in both peripheral blood and synovial tissue of RA patients (19, 20). These intermediate monocytes secrete pro-inflammatory cytokines, such as tumor necrosis factor-α (TNFα), interleukine-1β (IL-1β), and IL-6, and they can differentiate into pro-inflammatory M1 macrophages, contributing to the local synovial inflammation (**Figure 1**) (21).

RA intermediate monocytes are characterized by an increased expression of HLA-DR compared to the other two monocyte subsets, and this increased expression seems to determine a high production of TNFα (15, 19, 22). In addition, HLA-DR^+^ intermediate monocytes express high level of the costimulatory molecules CD80 and CD86 promoting the induction of IL17^+^CD4^+^ T cells (**Figure 1**) (15).

Therefore, in the peripheral blood and synovial fluid of RA patients, the predominance of intermediate monocytes seems to suggest their functional role in modulating Th17 cell responses through the production of IL-12, which stimulates CD4^+^ Th1 cell polarization, and the release of IL-6, IL-1β, and IL-23 (15). These cytokines drive Th17 cell polarization and the release of IL-17 by CD4^+^ T cells (15, 23).

As described in a previous study, the intermediate monocyte subset is the major subset to undergo differentiation into pro-inflammatory M1 macrophages (**Figure 1**) (24). Together with classical monocytes, the intermediate monocytes express Toll-like receptor-2 (TLR2) on their surface membrane in both peripheral blood and synovial tissue of RA patients. However, compared to classical monocytes, intermediate monocytes highly express TLR2, which activates the signaling pathway responsible for the production of the pro-inflammatory cytokines IL-1β, IL-6, and TNFα (25, 26).

Conversely, even if non-classical monocytes seem to participate in the early inflammatory response, they differentiate into resident M2 macrophages taking part in the resolution of inflammation (**Figure 1**) (27).

As is well-demonstrated, monocytes are essential players in the pathology of several inflammatory diseases, including RA, in which these cells are one of the two major contributors to the damage at synovial tissue level, together with macrophages (28). This fundamental role of monocytes is also related to their plasticity, which is also achieved by a highly responsive epigenome: this epigenomic plasticity of monocytes is determined by the occurrence of relevant DNA methylation changes (28).

Several studies revealed how the high expression levels of *de novo* DNA methyltransferase 3A (DNMT3A) and the methylcytosine dioxygenase ten–eleven traslocation-2 (TET2) in monocytes are essential for the differentiation and activation of these cells during inflammatory responses, suggesting how DNA methylation represents the major epigenetic mechanism that potentially reflects the influence of disease-associated inflammation in monocytes (29, 30).

The important role of methylation in monocyte pathophysiology is highlighted by a recent study, which demonstrated a difference in DNA methylation profiling between monocytes isolated from RA patients and healthy subjects: the study revealed how RA monocytes are characterized by hypermethylated CpG sites related to several genes, including IFN and TNF, suggesting a potential implication of these cytokines and their signaling pathways in the acquisition of a further aberrant DNA methylation signature in RA patients (28).

Therefore, in RA patients, the high percentage of monocytes, primarily belonging to the intermediate subset, and their increased DNA methylation are linked to the inflammatory environment in the blood, correlating with the high disease activity (evaluated by 28-joint Disease Activity Scale—DAS28), serum level of C-reactive protein (CRP), and erythrocyte sedimentation rate (ESR) (28). All these observations suggest a role of monocytes as additional biomarker for high disease activity in RA patients (31).

Moreover, circulating RA monocytes also express high levels of several chemokines, including CCR7, which interact with CCL19; the upregulation of CCR7/CCL19 correlates with disease activity (DAS28) and the radiographic progression of joint damage (32).

## Macrophages: Polarization and Signaling Pathways Involved in RA

Macrophages were described for the first time in 1882 by Metchnikov as the “big eater” of the immune system and represent the frontier soldiers of immune system, thanks to their capability to recognize, engulf, and destroy pathogens through the activation of TLRs and the production of pro- and anti-inflammatory mediators (33, 34).

As APCs, macrophages contribute to induce a Th1- or Th2-mediated immune response through the presentation of non-self-antigens to naive T cells and the release of cytokines and growth factors, confirming that their interplay with T lymphocytes represents a vital check point for T-cell maturation; this is a fundamental function in the regulation of inflammation and in the maintenance of homeostasis (35, 36).

Indeed, plasticity is a key feature also of macrophages, which are capable of presenting heterogeneous phenotypes creating various subpopulations; therefore, these cells are not only involved in the propagation of inflammation but also in its resolution, depending on their activation state (M1 or M2) (37). Therefore, it is becoming increasingly apparent that M1 and M2 phenotypes represent the extremes of a macrophage activated spectrum, which is characterized by the presence of “intermediate” phenotypes involved in the immuno-regulation or in tissue repair and defined by different metabolic pathways, surface markers, and cytokine production (37–40).

Due to the advanced research, science has made unbelievable progress during the past years, shedding light on the role of these cells in the immune response that characterizes RA.

**Figure 2:**
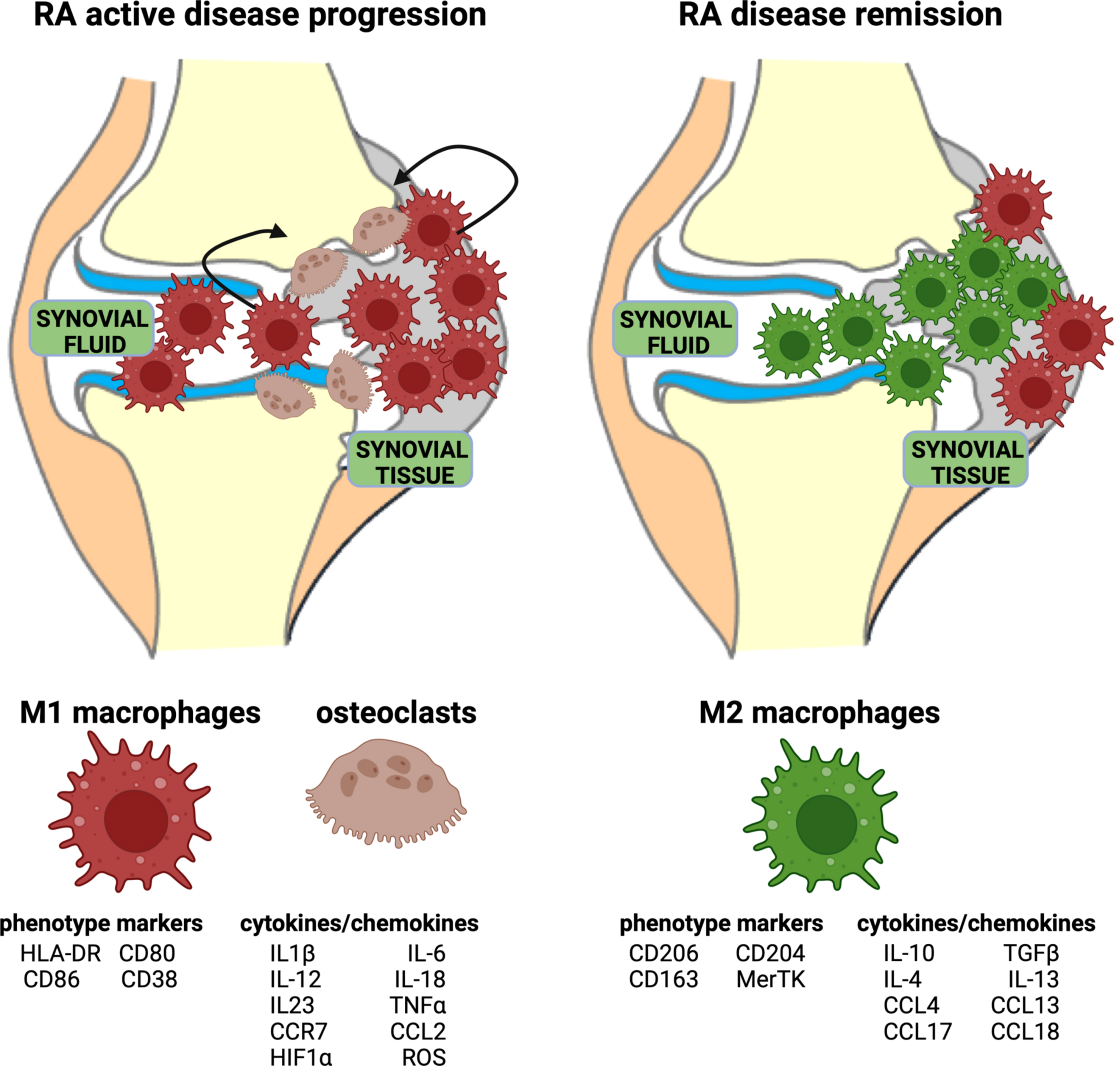
Representation of acute RA inflammation and remission. Acute RA inflammatory phase is characterized by an imbalance in M1–M2 ratio in synovial fluid and tissue. This phase is dominated by a higher percentage of pro-inflammatory M1 macrophages, which display specific phenotype markers and release cytokines/chemokines. Moreover, the activation of osteoclasts contributes to bone erosion. RA disease remission is characterized by a high percentage of anti-inflammatory M2 macrophages, which display specific phenotype markers and release anti-inflammatory cytokines/chemokines.

In RA, the inflammatory process is mediated and sustained by M1 macrophages both in peripheral blood and in synovial tissue (**Figure 2**). Indeed, M1 macrophages are pro-inflammatory cells characterized by the high expression of major histocompatibility complex (MHC) class II, CD80, CD86, CD38, and TLR4, and the secretion of pro-inflammatory cytokines, primarily IL-1β, IL-6, and TNFα, and chemokines, such as CCR7 (**Figure 2**) (35, 41).

Their prompt production of inflammatory cytokines stimulates the immune system enabling an efficient pathogen eradication. When self-tolerance is lost, inflammation persists evolving to a chronic maladaptive immune response. CD80/CD86 are costimulatory molecules present on these macrophages (among other cells) in response to activating signals finalized to pathogen suppression; these surface proteins bind to CD28 on naive T cells increasing sensitivity to T-cell receptor (TCR) stimulation and T-cell survival (42).

TLRs belong to a heterogenous receptor family distributed on the cell membrane or cytosol of APCs, including macrophages, natural killers, lymphocytes, endothelial and epithelial cells, and fibroblasts (43).

TLRs are one of the most ancient immunity tolls for host defense against infection recognizing pathogen-associated molecular patterns (PAMPs), and TLR2 and TLR4 are primarily involved in pathogen recognition (44).

**Figure 3:**
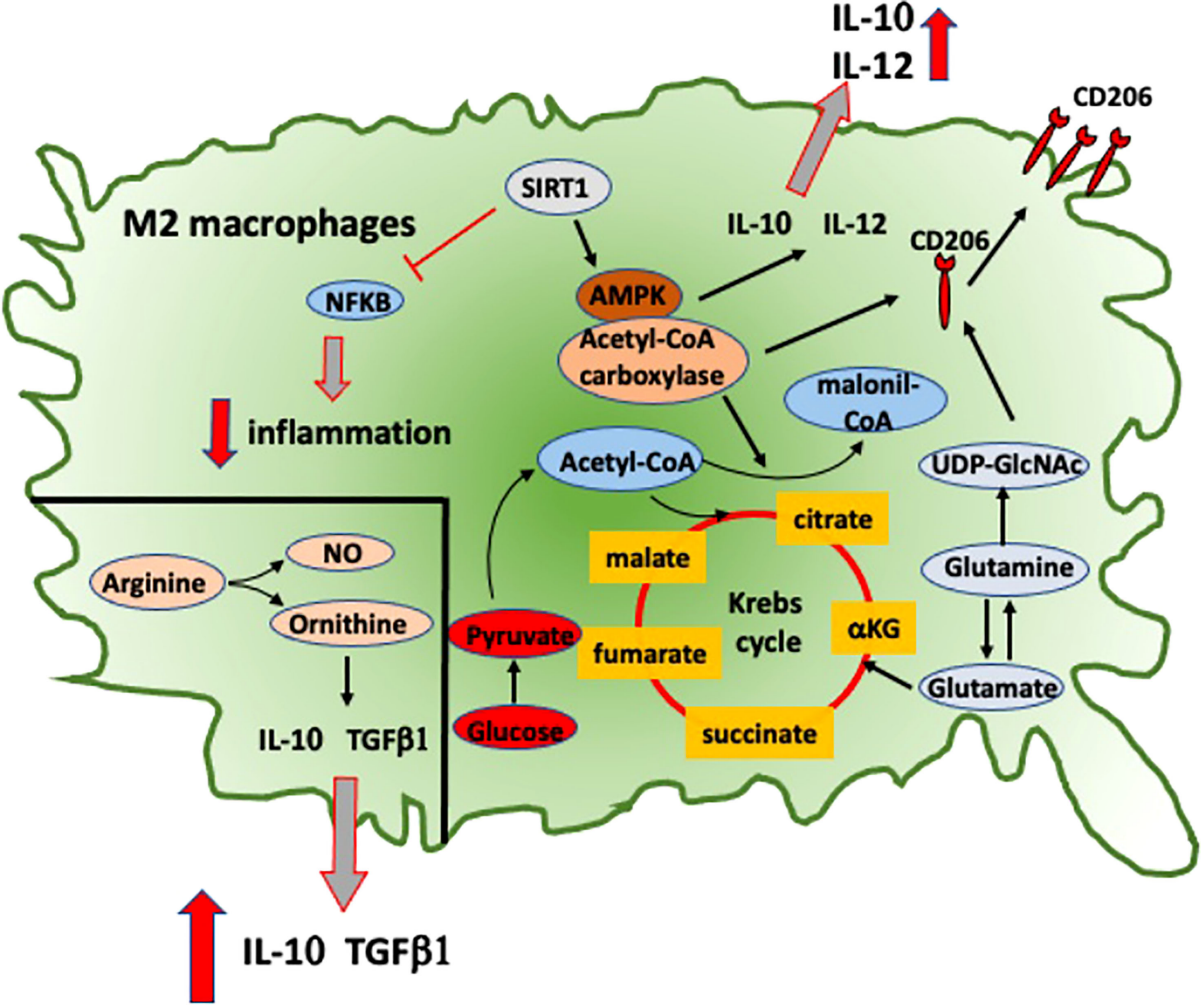
Intracellular signaling and metabolic pathways activated into RA anti-inflammatory M2 macrophages. Metabolic pathways activated in M2 macrophages that contribute to their anti-inflammatory role in RA. NFkB, nuclear factor-kB; SIRT1, sirtuin-1; AMPK, adenosine monophosphate-activated protein kinase; IL-10, interleukine-10; IL-12, interleukine-12; TGFβ1, transforming growth factor-β1; NO, nitric oxide; CD206, mannose receptor-1; UDP-GlcNAc, uridine diphosphate N-acetylglucosamine.

Moreover, the expression of TLR4 on macrophages permits to recognize endogenous ligands relevant in RA, such as native articular proteins and citrullinated peptides, and subsequently induces intracellular signal transduction finalized to a prompt expression of pro-inflammatory genes through the activation of nuclear factor kappa B (NF-kB) signaling pathway (33, 45): in fact, the activation of TLR4-induced NF-kB signaling pathway mediates the pro-inflammatory activity of M1 macrophages through the production and release of IL-6, TNFα, and IL-1β in monocyte-derived and synovial macrophages obtained from RA patients (**Figure 3**) (46, 47).

The polarization of macrophages toward an M1 phenotype can be induced by several pro-inflammatory stimuli, including the activation of IRF5 expression (48).

The upregulation of IRF5 activates the intracellular signaling pathway, which induces the transcription of several subunits of IL-12 and the repression of IL-10, with the subsequent induction of Th17 differentiation of T cells (48). M1 macrophages also express high levels of IL-15, which promotes MHC class II overexpression and SOCS3 suppression, contributing to the activation of the proliferation of CD4^+^T cells (49).

In RA, another important pathway linked with M1 macrophage-induced inflammation involves the activation of stress-activated protein kinases/mitogen-activated protein kinases (SAPK/MAPK) and Janus kinase/signal transducer and activators of transcription (JAK/STAT), which are activated by pro-inflammatory cytokines and promote both proliferation and survival of macrophages (**Figure 3**) (50). TNFα, IL-1β, and IL-6 also promote the activation of MAPK signaling pathways through the phosphorylation of ERK1/2, JNK, and p38 kinase in synovial cells derived from patients with chronic RA (**Figure 3**) (51, 52).

In the contest of different biological activities of macrophages that contribute to tissue homeostasis and disease pathogenesis, an interesting macrophage subset, called arthritis-associated osteoclastogenic macrophages (AtoMs), was recently identified in the synovial fluid and tissue of RA patients (53).

These macrophages are characterized as CX3CR1^+^HLA-DR^high^CD11c^+^CD86^+^ cells, and they have a high osteoclastogenic potential (53). CX3 chemokine receptor-1 (CX3CR1) is a fractalkine receptor and marker of monocyte-lineage cells including a population of osteoclast precursor in the bone marrow under homeostatic condition, and it is also an osteoclast precursor marker in inflamed synovium (53).

Nevertheless, the expression of CD11c and MHC class II implies that AtoMs may share functional characteristics of both macrophages and dendritic cells. Of note, the expression of CD80 and CD86 indicates that these cells may be involved in antigen presentation in local foci of arthritic joints.

Moreover, together with their capability to differentiate into osteoclasts, AtoMs are efficient in inducing the activation of TNF-producing CD4^+^T cells, contributing to the amplification of inflammation and bone destruction (53, 54).

These cells were also identified in a collagen-induced arthritis (CIA) mouse model, where their differentiation into osteoclasts seems to be mediated by the activation of receptor activator of NF-kB ligand (RANKL) signaling pathways and boosted by TNFα stimulation (53). This pathway involves the activation of the transcription factor Forkhead box M1 (FoxM1), whose inhibition blocks the differentiation of AtoMs into osteoclasts attenuating their inflammatory cytokine production in the synovium and reduces the articular bone erosion (53).

The “anti-inflammatory” M2 macrophages are phenotypically characterized by the expression of surface markers including macrophage scavenger receptors (CD163, CD204), mannose receptor-1 (CD206), and the MER proto-oncogene, tyrosine kinase (MerTK) (**Figure 2**). To fulfill their main role in tissue homeostasis preservation, these so-called “alternative activated” macrophages support proliferation, wound healing, and angiogenesis, and they mitigate inflammatory process. M2 macrophages are responsible for apoptotic cell clearance, production of extracellular matrix (ECM) components, and angiogenic and chemotactic factors (55, 56).

Additionally, IL-10 and TGFβ are molecules endogenously produced by M2 macrophages shifting the immune activation toward a tissue repair pattern (**Figure 2**) (55, 56). CD163 is a hemoglobin scavenger soluble or membrane-bound receptor highly expressed in resident tissue macrophages, which contributes to the anti-inflammatory local response lowering hemoglobin levels and promoting inflammation-resolving heme metabolites (57, 58).

CD206 is a mannose scavenger receptor mainly present in M2 macrophages and dendritic cells, known to be involved in collagen internalization and degradation (59). MerTK is a tumor-associated macrophage (TAM) receptor predominantly expressed in M2 macrophages during immunomodulation processes (60, 61). Through the interaction with the bridging ligands Gas6 and protein S, MerTK recognizes apoptotic cells facilitating their phagocytosis; this physiological process of clearance is fundamental for the maintenance of immune tolerance (60–63).

Moreover, MerTK-induced Gas6 expression amplifies IL-10 production reinforcing an M2 positive feedback (64). Recent data have shown a significant correlation in RA patients between the low relative proportion of MerTK^+^ to MerTK^−^ synovial tissue macrophages with disease flare upon drug withdrawal, suggesting a potential role of this molecule as biomarker (65). In RA macrophages, a signaling pathway described to promote the induction of M2 polarization is the adenosine-monophosphate-activated protein kinase (AMPK)/a-acetyl-CoA carboxylase, which promotes the upregulation of macrophage-derived chemokine (MDC), CD206, and IL-10 (**Figure 3**) (66).

This pathway is induced by sirtuin-1, which downregulates the pro-inflammatory IL-12, CCL2, and iNOS through the inhibition of NF-kB signaling pathway and promotes the polarization toward an anti-inflammatory M2 phenotype in cultured macrophages obtained from RA patients and CIA mouse model (**Figure 3**) (66).

**Figure 4:**
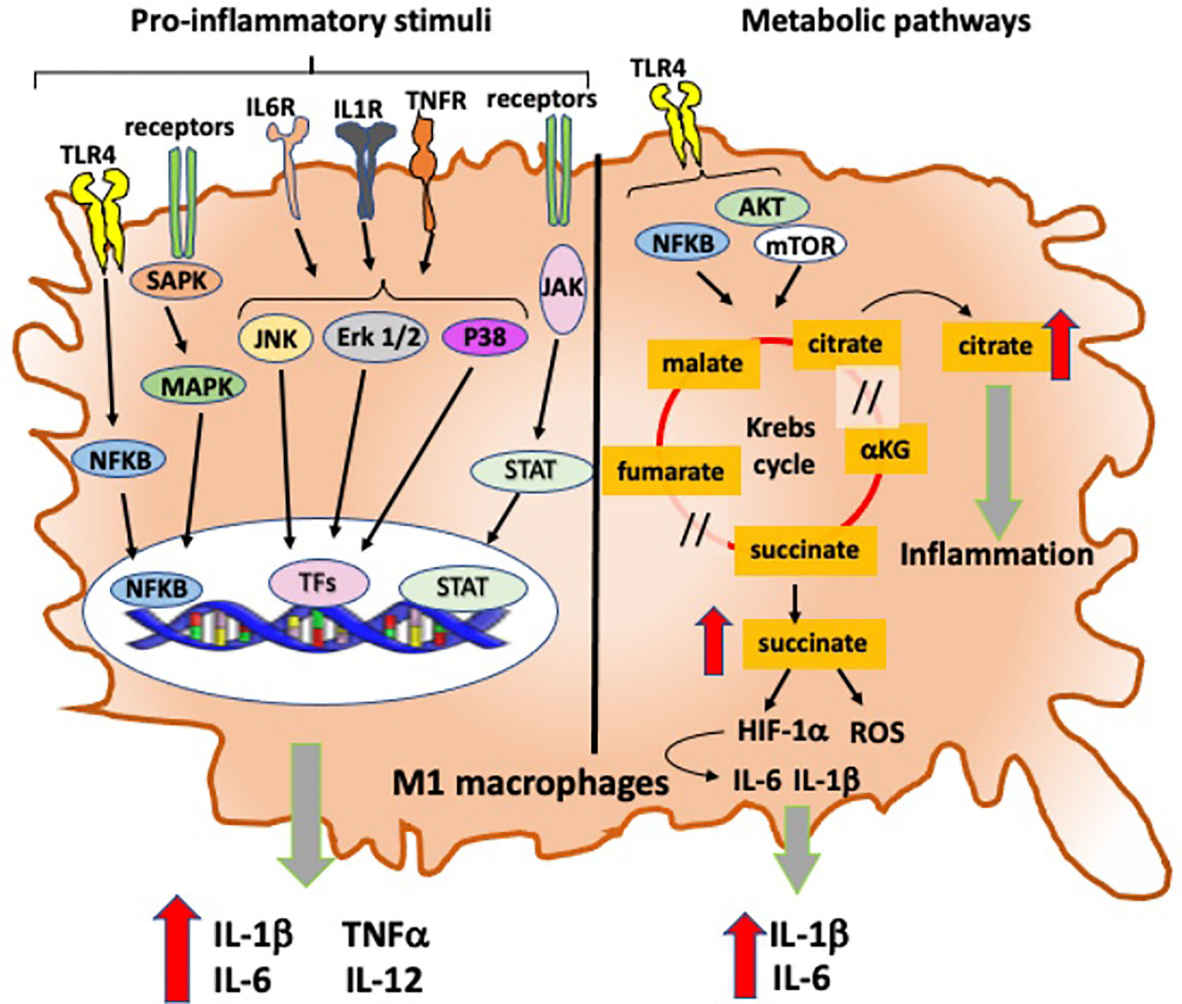
Intracellular signaling and metabolic pathways activated into RA pro-inflammatory M1 macrophages. Intracellular signaling and metabolic pathways activated in M1 macrophages that contribute to their pro-inflammatory role in the inflammatory process in RA. TLR4, Toll-like receptor 4; IL-6R, interleukine-6 receptor; IL-1βR, interleukine-1β receptor; TNFR, tumor necrosis factor receptor; NFkB, nuclear factor-kB; SAPK: stress-activated protein kinases; MAPK, mitogen-activated protein kinases; JAK, Janus kinase; STAT, signal transducer and activators of transcription; Erk1/2, extracellular signal-regulated protein kinases 1 and 2; JNK, Jun N-terminal kinase; TFs, transcription factors; AKT, protein kinase B; mTOR, mechanistic target of rapamycin; HIF-1α, hypoxia-inducible factor-1α; ROS, reactive oxygen species; /, break in Krebs cycle.

From a metabolic point of view, M1 and M2 macrophages show opposed metabolic profiles: M1 macrophages use preferentially aerobic glycolysis, while M2 macrophages relay on oxidative phosphorylation (**Figures 3**, **4**) (67). Therefore, during articular inflammation, synovial “pannus” formation and the presence of a hypoxic inflammatory environment drastically increase glycolytic activity in macrophages, which are polarized towards a M1 phenotype (**Figure 4**). Indeed, M1 cells more than other cell populations commonly present in synovial inflammatory tissues are responsible for cartilage damage (**Figure 2**) (68).

In inflamed joints, oxygen levels rapidly drop, while a raise in hypoxia factor 1α (HIF-1α) and reactive oxygen species (ROS) production occurs followed by the activation of inflammatory genes (IL-1β and IL-6), which promotes a massive oxidative tissue damage (**Figure 4**) (69). Additional elements may actively contribute to macrophages metabolic switch: for example, TLR4 activates aerobic glycolysis finalized to provide sufficient bioenergetic resources to support cell mature state (**Figure 4**) (70).

Moreover, succinate is a transformation product of glycolysis highly present in lipopolysaccharide (LPS)-activated M1 macrophages and able to stabilize HIF-1α and influences IL-1β expression (**Figure 4**) (71).

Ornithine and nitric oxide (NO) are the most characteristic molecules of macrophage polarization toward M1 or M2 active state, respectively. Both of these molecules are metabolites obtained through L-arginine cleavage. Ornithine promotes cell proliferation, tissue healing, and fibrosis through the deposition of polyamines and collagen. NO instead inhibits cell proliferation and a raise in IL-12/23 and IL-18 levels (**Figure 3**) (71).

Based on these observations, a metabolic reprogramming through the inhibition of glycolysis seems to modulate the polarization of macrophages from an M1 to an M2 phenotype: the glycolysis inhibitor 2-deoxyglucose ameliorates adjuvant-induced arthritis by regulating macrophage polarization in an AMPK-dependent manner (**Figure 3**) (72).

## Effects of M1 and M2 Macrophages in RA Synovitis

Synovial tissue is the major district of joint inflammation in RA patients, and the persistent chronic synovitis leads to an irreversible damage of cartilage and bone (73). The specialized structure of synovium is composed of two layers: the lining layer, which is populated by macrophages and FLSs, and a sublining layer constituted by vascularized connective tissue (74).

The synovial lining layer is a protective barrier, and synovial fluid is vital for physiological motion, maintaining cartilage and joints well hydrated. The absence of an epithelial basement membrane in the synovial lining contributes to its permeability and the diffusion of different compounds (73). Macrophages of the lining layer are resident cells involved in the maintenance of tissue homeostasis; these cells express CX3CR1, forming a protective tight-junction cell layer that avoids the infiltration of inflammatory cells responsible for arthritis development (75).

In the setting of synovitis, synovial tissue cellularity rises, and synovial thickening is commonly reported as radiographic feature. Moreover, most resident macrophages are still characterized by the expression of CD206, MerTK, and T-cell immunoglobulin and mucin domain containing 4 (TIMD-4) (75, 76).

Synovial macrophages along with infiltrating monocytes-derived macrophages are fundamental cells in the initiation and chronicity of RA synovitis through their capability to orchestrate the immune response releasing cytokines and enzymes involved in the inflammatory cascade, which in turn activate osteoclasts and fibroblasts, leading to joint destruction and disease perpetuation (**Figure 2**) (35, 77, 78).

These macrophages express TLRs, primarily TLR2, and activate local danger signals and modulate their activity (79).

In RA synovial tissue, the interaction between activated M1 macrophages and Th1 cells fosters the production of several pro-inflammatory mediators, including IL-1β, IL-6, TNFα, IL-23, CXCLs, and CCLs; this crosstalk is mediated first by MHC class II and secondarily by costimulatory molecules CD80/CD86, which are overexpressed in RA M1 macrophages (**Figure 2**) (77, 80, 81). In the early-stage of RA, these mediators contribute to the recall and activation of monocyte-derived macrophages from peripheral blood into synovial tissue promoting and sustaining inflammation (82).

During disease progression, the synovial tissue responds to inflammatory insults with a maladaptive wound healing undergoing profound changes: inflammatory and mesenchymal cells infiltration, inner-layer hyperplasia, neovascularization, and pannus formation resulting in cartilage destruction.

As previously discussed, CCR7 signaling pathway was demonstrated to induce monocyte migration and M1 polarization: in fact, CCL21/CCR7 signaling mediates the migration of CD14^+^CD86^+^ monocytes, which polarize into M1 macrophages with a consequential pro-inflammatory cytokine production, primarily IL-6 and IL-23 (83).

RA macrophages and primarily pro-inflammatory M1 macrophages are characterized by a high expression of CCR7, and these CCR7-expressing macrophages induce and amplify the differentiation of Th17 cells (83). Moreover, the activation of CCL21/CCR7 signaling pathway in these macrophages determines their differentiation into osteoclasts in a process that involves the induction of Th17 polarization (83). CCR7 expression on monocytes is enhanced by IFNγ and TNFα, whereas its drastic reduction has been observed in the presence of IL-4, a typical Th2/M2 mediator (83).

Together with CCL21, IL-23 is another important cytokine mainly secreted by activated macrophages in the synovial tissue, which induces the differentiation of αβ CD4^+^ naive T cells into Th17 cells (23). These CD4^+^ T cells are the major producers of IL-17, which characterize the synovial compartment of RA patients and contribute to the pathogenesis of the disease (23). This cytokine interacts with its receptor on the surface membrane of several cell types, including monocytes/macrophages, activating several intracellular signaling pathways involved in the inflammation, such as those mediated by Erk1/2, JNK, p38, STATs, and JAK activation (23). As is well-demonstrated, the synergic effect of IL-17 and TNFα induces the production of pro-inflammatory mediators by macrophages, including IL-6, IL-1β, and matrix metalloproteinases (MMPs) that contribute to the progression of an early inflammation toward a chronic arthritis (84).

Moreover, these inflammatory macrophages are involved in the turnover of connective tissue and erosion of articular surface through their production and release of MMPs (85). The massive release of pro-inflammatory cytokines and chemokines determines a drastic change in the synovial microenvironment and allows an efficient activation of cytotoxic cells (85).

As a hallmark of inflammation, the abundant presence of macrophages (M1) in RA synovitis reflects disease activity, and therefore, their depletion at the level of target organ may be a good biomarker of therapeutic response (**Figure 2**) (86).

Many studies have confirmed the different origin of resident macrophages and monocyte-derived macrophages (86).

In the synovial tissue of RA patients and healthy subjects, resident macrophages are identified as CD68- and CD163-positive cells, able to proliferate and maintain themselves locally: these cells remain relatively quiescent, while they are activated during disease flares (75, 87). CD68 was shown to bind oxidized low-density lipoprotein and to be involved in the cell–cell interaction. In the synovial sublining, changes in the number of CD68^+^ macrophage correlates with clinical outcomes evaluated using DAS28, representing a possible further reliable biomarker of therapeutic efficacy (87, 88).

In a recent study involving long-standing RA patients, the analysis of transcriptome profile of highly inflamed synovial tissue demonstrated the upregulation of transcripts related to the signaling pathways mediated by TLR, TNF, IFN, and IL-6 receptors and related to chemotactic and inflammatory processes, overlapping with those monocyte/macrophage patterns activated by bacterial and fungal pathogens, such as LPS (79).

As is well-demonstrated, *in vitro* stimulation of circulating human monocytes with LPS induces their differentiation and polarization into a pro-inflamatory M1 phenotype, characterized by the expression of specific surface markers CD80, CD86, HL-DR, TLR2, and 4, and the release of IL-1β, TNFα, and IL-6 (**Figure 2**) (42, 89). In RA synovitis, the best represented and upregulated genes and the secreted proteins are those correlated to M1 macrophages (79).

Moreover, among these secreted proteins, sCD14, S100A8/A9, S100P, LBP, CXCL13, MMP-3, and CCL18 showed a good correlation between their concentration and the DAS28/ESR (79).

In the synovial tissue and fluid of RA patients, CD86^high^AtoMs characterized by an increased FoxM1 gene expression show a high osteoclastogenic potential compared to CD86^low^AtoMs, contributing to the inflammatory process and bone erosion in RA (53).

Conversely, MerTK^+^CD206^+^ synovial tissue macrophages (STMs) are highly expressed in RA patients during the remission state (**Figure 2**) (64). MerTK^−^CD206^−^ STMs are the main source of pro-inflammatory cytokines in synovitis and the cell–cell interactions between macrophages and fibroblasts (64).

MerTK is a member of transmembrane receptor tyrosine kinase family, expressed on the surface membrane of macrophages and dendritic cells. After activation by its ligand Gas6 and protein S, MerTK plays an anti-inflammatory action inducing the phagocytosis of apoptotic cells, a key process for tissue repair and the maintenance of tissue homeostasis (90).

In human synovial tissue, MerTK^+^ synovial macrophages are characterized by a specific regulatory signature depending on the disease state (healthy, active RA, or remission): in particular, RA patients who underwent remission show the upregulation of the genes encoding for the transcription factors Krüppel-like factor 2 (KLF2), KLF4, nuclear receptor subfamily 4 group A member 1 (NR4A1), NR4A2, or the dual-specificity phosphatase1 (DUSP1), representing negative regulators of inflammation that actively participate to restore tissue homeostasis, through lipid mediators such as resolvins (64).

## Old and New Therapeutic Strategies Inducing the M1–M2 Polarization and Future Perspectives

In RA patients, the high expression of pro-inflammatory molecules induces monocytes, primarily those belonging to the intermediate subset, to migrate to synovial tissue and differentiate into M1 macrophages.

It is evident that the increased presence of activated pro-inflammatory macrophages in synovial tissue is considered an early hallmark of RA, and it is correlated with the higher proportion of M1 macrophages compared to M2 macrophages (**Figure 2**) (91, 92).

Therefore, an early inhibition of macrophages activation may be considered as an effective and well-tolerated therapeutic strategy in the management of RA (93, 94).

As is well-demonstrated, a prompt diagnosis followed by an early treatment is mandatory to prevent debilitating bone erosions, functional decline, and premature mortality in RA patients (95). Achieving early remission within the “therapeutic window of opportunity” determines better clinical outcomes and consequently future treatment avoidance (96).

Conversely, a delay in starting treatment results in prolonged symptom duration and poorer outcomes (97).

The identification of specific biochemical markers reflecting macrophage populations could be a useful tool to identify disease activation state and represent possible targets for RA treatment, such as the aforementioned MerTK (58).

Interestingly, in RA patients in disease remission, a high presence of MerTK^+^CD206^+^CD163^+^ M2 macrophages has been detected in the synovial tissue, where they formed a tight lining layer; the increased presence of these cells was negatively correlated with DAS28/CRP, synovial hypertrophy, and vasculitis (64).

Of note, the presence of these cells was also observed in healthy synovial tissues. Conversely, active RA patients were characterized by the presence of MerTK^-^CD206^-^macrophages in the lining layer of the synovial tissue (64).

Interestingly, this study confirmed that in RA patients where it was possible to taper and then discontinue biological treatment before the investigation of synovial tissue macrophages, the disease remission was maintained in those patients characterized by a high percentage and proportion of MerTK^+^CD206^+^ macrophages (M2 macrophages) (**Figure 2**); conversely, in those RA patients who flared after biological treatment discontinuation, the percentage of these M2 macrophages was lower (64).

These results indicate that MerTK^+^ macrophages showing an M2 phenotype seem to characterize the synovial tissue of RA patients under disease remission and healthy subjects (**Figure 2**): these cells produce lipid mediators implicated in the resolution of inflammation, and they overexpress transcription factors implicated in the control of local immune responses and homeostasis.

Therefore, based on this new evidence, the induction of the MerTK signaling pathway might be considered a promising approach in driving disease remission in RA patients (63).

Interestingly, compelling evidence have demonstrated a positive correlation between glucocorticoid therapy and the augmented MerTK expression on monocyte-derived macrophages surface membrane, revealing an additional role of this therapeutic approach in RA flare attenuation.

Furthermore, cellular metabolic reprogramming could be an innovative therapeutic strategy to reduce M1 macrophage growth and alter inflammatory milieu in favor of anti-inflammatory M2/Th2 pathways, restoring the correct balance in the M1–M2 ratio (98).

In the last decades, RA treatment has significantly been changed, highlighting the pivotal role of treat-to target strategies aiming to a patient tailored therapy for a better control of disease activity. Therefore, the acknowledge of RA pathophysiology has been a crucial guide for the development of effective and safe treatments. About that, in the past years an increased number of biological disease-modifying anti-rheumatic drugs (bDMARDs) have been developed with a proven efficacy (99).

Indeed, starting with bDMARDs treatment at a very early stage can modify or even reverse disease progression thanks to their ability to interfere with biologic processes (96). Although these drugs are structurally unrelated and have different pharmacodynamic and pharmacokinetic properties, their clinical development has been largely overlapping (99).

Currently, no drugs are specific for macrophages in the treatment of RA, but their effects are directed to inhibit some aspects of macrophage activation, in particular the production of inflammatory cytokines, including TNFα, IL-1β, and IL-6: monoclonal antibodies or soluble receptors have been used for many years, but novel agents targeting these molecules seem to be more efficient in the treatment of inflammatory phase in RA (92).

TNF inhibitors (including infliximab, etanercept, adalimumab, golimumab, and certolizumab) bind to soluble and membrane-associated TNFα, inhibiting the activation of those intracellular signaling pathways involved in mediating pro-inflammatory properties, including NF-kB and RANK ligand (**Table 1**) (2).

**Table 1:** Targets, effect, and signaling pathways of biological disease-modifying anti-rheumatic drugs (bDMARDs).

Treatment	Target	M1–M2 shift contribution	Signaling	Reference
CTLA4-Ig (abatacept)	CD80/CD86	Downregulation of CD80, CD86, and TLR4 Upregulation of CD204, CD163 and CD206, MerTK	Inhibition of NFkB	(42) (46) (47)
TNF inhibitors (infliximab, etanercept, adalimumab, golimumab and certolizumab)	TNFα	Upregulation of IL-10, SOCS3, GAS6, CD16	Activation of STAT3 Inhibition of NFkB	(100)
Rituximab	Anti-CD20	Downregulation of CD40	–	(100)
Tocilizumab	Anti-IL-6R	Downregulation of CD40 Upregulation of CD206	–	(100)

Description of molecular targets, effect exerted on cells involved in the inflammatory process and signaling pathways modulated by biological disease-modifying anti-rheumatic drugs, such as CTLA4-Ig (abatacept), TNFα inhibitors (infliximab, etanercept, adalimumab, golimumab, certolizumab), anti-CD20 antibody (rituximab), and anti-IL-6 receptor antibody (anti-IL-6R, tocilizumab).

TLR4, toll-like receptor 4; CD204 and CD163, macrophage scavenger receptors; CD206, mannose receptor 1; MerTK, MER proto-oncogene, tyrosine kinase; TNF, tumor necrosis factor; IL-10, interleukine-10; SOCS3, suppressor of cytokine signaling 3; GAS6: growth arrest-specific 6; STAT3, signal transducer and activator of transcription 3.

Tocilizumab inhibits the IL-6-mediated inflammation through the interaction with IL-6 receptors, whereas the immune and pro-inflammatory action of IL-1β is contrasted by the inhibition of the binding with its receptors mediated by anakinra, a non-glycosylated recombinant form of the physiological IL-1 receptor antagonist (**Table 1**) (2, 100).

As matter of fact, in a recent study, the contribution of some bDMARDs, in particular anti-TNF agents, on the impact of pro-inflammatory M1 macrophages obtained from RA patients revealed their indirect capability to modulate the polarization of these cells toward an M2 phenotype (**Table 1**) (101).

The mechanism that promotes this polarization involves the activation of Gas6 and SOCS3 and the subsequent increase in IL-10, a process mediated by the induction of STAT3 signaling pathway (**Table 1**) (101).

Conversely, this effect in promoting the polarization from a M1 to an M2 phenotype seems not to be induced by the treatment with anti-IL-6 receptor and anti-CD20 agents, which do not determine the upregulation of M2 markers in cultured macrophages (**Table 1**) (101).

## The Recent Discovered Role of CTLA4-Ig (Abatacept) in Inducing the M1–M2 polarization

The capability of a selected bDMARDs to promote the polarization of pro-inflammatory M1 macrophages to an anti-inflammatory M2 phenotype was recently tested *in vitro* for the CTLA4-Ig fusion protein in cultured monocyte-derived macrophages obtained from RA patients (**Table 1**) (42). These RA monocyte-derived macrophages, which were characterized by a pro-inflammatory M1 phenotype, as demonstrated by their upregulation of CD80, CD86, and TLR4 gene expression, acquired an anti-inflammatory M2 phenotype after treatment with CTLA4-Ig. This polarization is determined by the downregulation of the gene expression of M1 phenotype markers and the upregulation of the gene and protein expression of M2 cell surface markers CD204, CD163, and CD206 and MerTK, suggesting also an increased induction of their phagocytic activity (**Table 1**) (42).

However, this important result was anticipated by the demonstration that the inhibition of the CD80-CD86/CD28 co-stimulatory signaling pathway by CTLA4-Ig generally contributes to downregulate several pro-inflammatory mediators involved in the inflammatory cascade of RA (**Table 1**) (102–105).

In fact, in RA patients, the treatment with abatacept significantly reduced serum levels of IL-6, IL-12, IL-1β, and soluble E-selectin and ICAM-1, together with the reduction in IFNγ and MMP-1/3 gene expression (**Table 1**) (102, 103). This reduction of these important inflammatory mediators determines an improvement of disease outcomes. Of note, several *in vitro* studies demonstrated the capability of CTLA4-Ig to block the differentiation of monocytes into osteoclasts, reducing the expression of CD80 and CD86, without affecting mature osteoclasts, the functions of which are important in terms of physiological bone homoeostasis and bone turnover (106–108). On the contrary, this physiological effect is not induced by other bDMARDs (106–108).

RA patients with an inadequate response to bDMARDs have a significant reduction in the composite score of DAS28/CRP level and the patient’s global assessment of disease activity after 12 weeks of treatment with abatacept (109).

More specifically, several *in vitro* studies highlighted the capability of CTLA4-Ig treatment to reduce the gene expression and release of pro-inflammatory cytokines IL-6, IL-1β, and TNFα directly interacting with CD86 on the surface membrane of APCs, primarily synovial macrophages and monocyte-derived macrophages isolated from RA patients (104, 105). This direct anti-inflammatory effect is mediated by the inhibition of NF-kB signaling pathway in a short time (**Table 1**) (46, 47).

## Concluding Remarks

In the pathogenesis of RA, monocytes and macrophages are fundamental mediators of the inflammatory process, contributing to the T-cell activation and production and release of pro-inflammatory cytokines and chemokines responsible for the migration of circulating cells to the synovial tissue and promoting an aberrant immune response that leads to the perpetuation of inflammation and bone erosion.

The development of this inflammatory environment is primarily due to an imbalance in M1–M2 monocytes/macrophages both in the peripheral blood and synovial tissue with a predominant presence of M1 macrophages, which also contribute to osteoclastogenesis in RA patients with active disease (14).

Conversely, the synovial tissue of RA patients under remission is characterized by a higher presence of M2 macrophages with a phagocytic activity compared to patients with active disease. Considering that the regulation of M1/M2 imbalance in favor of anti-inflammatory M2 macrophages might represent a therapeutic goal to restore tissue homeostasis, the identification of molecules that may promote M1/M2 polarization of RA macrophages may represent valuable therapeutic targets and could lead to the development of novel drugs.

Based on the newest acknowledgments concerning the therapeutic strategies currently used in clinical practice, the treatment inducing not only the downregulation of pro-inflammatory cytokines/chemokines but also the polarization from M1 into anti-inflammatory M2 macrophages might be an interesting approach to better control the aberrant inflammatory response in RA patients.

## Author Contributions

MC and SS conceptualized the argument of the review, collected the data, and wrote the manuscript. RC collected the data and wrote the manuscript. EG reviewed the manuscript for important intellectual content. All authors contributed to the article and approved the submitted version.

## Conflict of Interest

The authors declare that the research was conducted in the absence of any commercial or financial relationships that could be construed as a potential conflict of interest.

## Publisher’s Note

All claims expressed in this article are solely those of the authors and do not necessarily represent those of their affiliated organizations, or those of the publisher, the editors and the reviewers. Any product that may be evaluated in this article, or claim that may be made by its manufacturer, is not guaranteed or endorsed by the publisher.
